# Adult-Onset Still’s Disease in a 28-Year-Old Man From Ghana

**DOI:** 10.7759/cureus.18126

**Published:** 2021-09-20

**Authors:** Simon Kashfi, Sapna Sharma, Victoria Bengualid, Shorabh Sharma, Liliya Gandrabur

**Affiliations:** 1 Internal Medicine, City University of New York (CUNY) School of Medicine, New York City, USA; 2 Internal Medicine, Mahatma Gandhi Mission Institute of Health Sciences, Navi Mumbai, IND; 3 Infectious Diseases, St. Barnabas Hospital Health System, Bronx, USA; 4 Medicine, City University of New York (CUNY) School of Medicine, New York City, USA; 5 Internal Medicine, St. Barnabas Hospital Health System, Bronx, USA; 6 Rheumatology, St. Barnabas Hospital Health System, Bronx, USA

**Keywords:** interleukin (il)-6, systemic juvenile idiopathic arthritis, high ferritin, yamaguchi, adult onset still's disease (aosd)

## Abstract

Adult-onset Still’s disease is a rare, autoinflammatory disease characterized by spiking fevers, arthritis, salmon-colored skin rash, and leukocytosis. It has been compared to systemic juvenile idiopathic arthritis because of its similar features but is much rarer than its pediatric counterpart. It is usually treated with corticosteroids and disease-modifying anti-rheumatic drugs. However, those with refractory disease are candidates for one of many biological therapies. We present the case of a 28-year-old man who was successfully managed with first-line steroid therapy.

## Introduction

Adult-onset Still’s disease (AOSD) is a rare, autoinflammatory disease characterized by spiking fevers, arthritis/arthralgia, salmon-colored skin rash, and leukocytosis with neutrophilia >80% [1]. Neurologic manifestations have also been noted [2]. Prevalence is 1 to 34 cases per 1,000,000 people [3]. This disease was initially described by Bywaters in 1971 [4], is a diagnosis of exclusion, and the most popular and sensitive diagnostic criteria are the Yamaguchi criteria [5]. There are three phenotypes of the disease: monocyclic, polycyclic, and chronic [3]. The chronic form has the worst prognosis [1]. The recognition of AOSD is difficult because it mimics viral syndromes. One important biomarker is elevated ferritin, often several times the upper limit of normal [1,6,7]. Treatment for AOSD is stepwise. Typically, corticosteroids are the first line, followed by methotrexate as a disease-modifying anti-rheumatic drug (DMARD) [3]. Patients with refractory disease, defined as not well controlled with these first- and second-line agents, are candidates for biologic therapies. We present the case of a 28-year-old male who was found to have AOSD that was successfully managed with first-line steroid therapy.

## Case presentation

The patient is a 28-year-old male from Ghana with no medical history who presented to the emergency room with fevers, “on and off” generalized arthralgias, and morning stiffness for 1.5 years. This was associated with occasional swelling of the fingers “like sausages” that would resolve over the course of the day with movement and with acetaminophen or non-steroidal anti-inflammatory drugs. He did not report erythema of the joints. The pain was located in small joints (hands > feet), wrists, and knees. The patient also complained of anterior chest discomfort triggered by cough for 4-5 days prior to presentation. He reported subjective weight loss, decreased appetite, and fatigue for the last month, but denied night sweats and rash. He also denied oral ulcers and urethral discharge. He last traveled to Ghana two months before the presentation.

In triage, he was febrile with a temperature of 101.8 F and tachycardic at 128 beats per minute, with a blood pressure of 105/54 mmHg. Physical exam was notable for dehydrated oral mucosa, but otherwise unremarkable. No hepatosplenomegaly, nail, or joint changes were noted. Erythrocyte sedimentation rate (ESR) was 105 mm/hr. The patient was also found to have anemia, with a hemoglobin of 7.7 g/dL and a mean corpuscular volume of 63 fL. CT scan of the chest without contrast showed small right pleural effusion with bibasilar patchy airspace opacities concerning pneumonia. He also had axillary, mediastinal, and hilar lymphadenopathy concerning lymphoma, and a small pericardial effusion with mild cardiac enlargement. CT scan of the abdomen and pelvis was notable for hepatosplenomegaly, no retroperitoneal masses or lymphadenopathy, and numerous prominent bilateral inguinal lymph nodes. An echocardiogram showed minimal pericardial effusion, normal valves, and no vegetations. He was admitted to the general medical floor for further workup. The differential diagnosis at this time was large, including lymphoma, sarcoidosis, AOSD, Kikuchi disease, Whipple’s disease, IgG4-related disease, in addition to infectious causes such as histoplasmosis, tuberculosis, cryptococcosis, syphilis, Coxiella burnetii, and paracoccidioidomycosis.

On the floor, the patient had repeated spiking fevers, with temperature ranging from 97.4 to 102.9 F, while on antipyretic medicine (Table 1). The temperature was not continuously measured, and there are gaps from evening to morning (as per hospital norm), so it is difficult to determine a fever pattern. He was also tachycardic, with heart rates ranging from 106 to 131 beats per minute. The patient had diagnostic and rheumatologic workup as described in Tables 2, 3. 

**Table 1:** Time and temperature during the hospital stay

Time	Temperature (F)	Time	Temperature (F)
Day 1		Day 8	
16:51	101.8	6:00	100.3
23:43	102.8	16:00	97.6
Day 2		22:00	97.5
0:51	102.2	Day 9	
3:00	100.3	6:00	97.9
6:00	98.9	14:00	98.2
14:00	98	22:00	100.9
22:00	102.9	Day 10	
Day 3		6:00	99
6:00	101.7	14:00	98.1
14:00	97.4	22:00	101.1
22:00	99.8	23:00	98.4
Day 4		Day 11	
6:00	100.8	6:00	98.6
14:00	98.2	14:00	98.4
22:00	99	22:00	99.8
Day 5		Day 12	
6:00	101.4	6:00	98.7
11:44	97.9	14:00	98.3
14:00	98.3	22:00	99.1
22:00	100	Day 13	
Day 7		6:00	97.8
6:00	99.9	14:00	98.6
22:00	98.9		

**Table 2:** Initial diagnostic workup of the patient Normal ranges in parenthesis. WBC = White blood cell count, RBC = Red blood cell count, Hgb = Hemoglobin, AST = Aspartate aminotransferase, ALT = Alanine aminotransferase. Interpretation of hemoglobinopathy fractionation cascade: normal hemoglobin

Test	Result
WBC	9.6 (4.2-9.1 * 10^3/μL)
Neutrophils	72.7 (34.0-67.9%)
RBC	4.17 (4.63-6.08 * 10^6/μL)
Hemoglobin	7.7 (13.7-17.5 g/dL)
Hematocrit	27.0 (40.1-51.0%)
Mean corpuscular volume	63 (80.0-100.0 fL)
Platelets	239 (163-337 * 10^3/μL)
Iron saturation	8 (20-50%)
Total iron binding capacity	170 (240-450 μg/dL)
Ferritin	>7500 (12-300 ng/mL)
Hemoglobinopathy fractionation cascade	
Hgb A	97.8 (96.4-98.8%)
Hgb A2	2.2 (1.8-3.2%)
Hgb F	0.0 (0.0-2.0%)
Hgb S	0.0 (0.0%)
Total bilirubin	0.4 (0.3-1.2 mg/dL)
AST	121 (8-33 U/L)
ALT	44 (4-36 U/L)
Total protein	6.8 (6.0-8.5 gm/dL)
Albumin	1.9 (3.5-5.5 g/dL)
Serum protein electrophoresis	Small band of restricted mobility
Hepatitis C	Negative
Hepatitis B	Natural immunity
Hepatitis B	14 (60-170 μg/dL)
ESR	105 (0-22 mm/hr)
C-reactive protein (CRP)	20.56 (<10 mg/dL)
Lactate dehydrogenase (LDH)	1,096 (140-280 U/L)
Sputum culture	Positive for pseudomonas
Urinalysis	Normal

**Table 3:** Rheumatologic workup of the patient Normal ranges in parenthesis

Test	Result
Rheumatoid factor	Negative
Antinuclear antibody	Negative
Anti-double stranded DNA antibody	Negative
Anti-cyclic citrullinated peptide	Negative
Antineutrophil cytoplasmic antibody	Negative
Cryoglobulin	Negative
Angiotensin-converting enzyme level	73 (14-82 U/L)
Complement C3 level	191 (82-167 mg/dL)
Complement C4 level	37 (12-38 mg/dL)
IL-6 level	32.3 (0.0-13.0 pg/mL)
Thick and thin blood smear	Negative for parasites
Epstein Barr Virus IgM	<36.0 (0.0-35.9 U/mL)
Cryptococcus antigen	Negative
Tuberculosis testing	
Acid-Fast Bacilli smear and culture	Negative 3x
Quantiferon	Indeterminate 2x

Axillary lymph node biopsy was performed and the pathology report supported a reactive etiology. Axillary lymph node flow cytometry was also normal, without evidence of B- or T-cell lymphoma. The patient was ultimately diagnosed with AOSD and was started on 60mg of prednisone once daily, with a resolution of his fevers during the last three days of his hospital stay. He was discharged with 50mg of prednisone once daily for three weeks. In outpatient follow-up, he was doing well, with decreased joint pains, especially in the small joints of the hand. His anemia improved, with hemoglobin of 10 g/dL. Ferritin decreased to 400 ng/mL, LDH decreased to 282 U/L, and CRP decreased to 6.32 mg/L. Repeat CT scan of the abdomen and pelvis showed decreasing splenomegaly. The patient will likely require a DMARD for further treatment after the completion of his taper.

## Discussion

The most common diagnostic criteria to help guide the diagnosis is the Yamaguchi criteria (Table 4). It has a sensitivity of 93.5% [3]. The criteria are described in Table 4, and diagnosis requires at least 5 total criteria, with a minimum of two major criteria. Our patient met two major and four minor criteria, and it is likely that he had the polycyclic variant. He met the major criteria of fever and arthralgia, and the minor criteria of lymphadenopathy, hepatosplenomegaly, abnormal liver function tests, and negative antinuclear antibody and rheumatoid factor. One caveat to the Yamaguchi criteria is its age - the criteria was made in 1992 when little was known about the pathophysiology of the disease. Specifically, the exclusion criteria of “infection” have been challenged [1,3,8]. Many patients with AOSD have been found to have a concurrent infection, meeting exclusion criteria. The infection is hypothesized to be an “antigenic driver” [9] that causes aberrant production of proinflammatory cytokines in a dysregulated immune response [6]. Although respiratory cultures were positive for pseudomonas, this was not believed to be the cause of his symptoms. Additionally, he was negative for acute Epstein Barr Virus infection. It has been shown that patients with AOSD have high levels of IL-18, IL-6, and Il-1B and that IL-18 may be a biomarker to diagnose and assess disease severity in the future [1]. It remains to be seen whether another, more modern criteria will become the standard in the future. Two other criteria exist, Fautrel criteria and Cush criteria, though these both are less sensitive than the Yamaguchi criteria [3].

**Table 4:** Yamaguchi criteria depicting common diagnostic criteria to help guide the diagnosis

Major criteria	Minor criteria	Exclusion criteria
Fever > 102.2 F > 1 week	Sore throat	Infection
Arthralgia or arthritis > 2 weeks	Lymphadenopathy	Malignancy
Typical rash	Hepatomegaly/splenomegaly	Other rheumatic disease
WBC > 10,000/mL (> 80% Neutrophils)	Abnormal liver function tests	
	Negative antinuclear antibody and rheumatoid factor	

AOSD has been compared to other autoinflammatory syndromes, namely systemic juvenile idiopathic arthritis (sJIA). Jamilloux et al. propose that AOSD and sJIA are on a continuum because they have similar symptoms [6,10]. They both feature fever, skin rash, myalgia, and sore throat. Additionally, there are mono and polyarticular variants to juvenile idiopathic arthritis, similar to the mono and polycyclic variants of AOSD. The two are also similar because seasonality has been described. This also provides support for the infection hypothesis. There are differences between the two, however. First, AOSD affects women more than men, while sJIA has an almost equal ratio between boys and girls. One possible explanation for this is differences in hormone levels pre and post-puberty [6]. Additionally, the incidence of sJIA is much higher than AOSD. This is thought to be due to greater exposure to infectious agents in childhood. Other autoinflammatory diseases that AOSD has been compared to are cryopyrin-associated periodic syndromes (CAPS), TNF receptor-associated periodic syndromes (TRAPS), and familial Mediterranean fever (FMF). CAPS and TRAPS are single gene defect disorders, while FMF is confined geographically to the Mediterranean. AOSD is not caused by a single gene defect, nor is it confined to a single geographic region.

AOSD has also been grouped with other hyperferritinemic syndromes, namely macrophage activation syndrome (MAS), catastrophic antiphospholipid syndrome, and septic shock [1,3]. Ferritin is an acute-phase reactant that increases in response to infection and is produced by hepatic macrophages. It is now hypothesized to be a proinflammatory cytokine. Of note, the Yamaguchi criteria combined with glycosylated ferritin <20% increase sensitivity to 98.2% [1]. Normal levels of glycosylated ferritin are >50%, but they decrease due to increased production.

The first-line treatment for AOSD is corticosteroids, causing a clinical response in 60% of patients [3]. Interestingly, 45% of patients cannot be weaned off steroids due to recurrence, and thus become steroid dependent. In these patients, and in those who are unresponsive, the DMARD methotrexate is the second-line drug. It is used for its steroid-sparing effect. In those with refractory disease, biologic therapies are indicated due to their anti-inflammatory effects. However, there is not a clear frontrunner for best biologic, as many proinflammatory markers are implicated in the pathogenesis of AOSD. Tocilizumab, an IL-6 receptor antagonist, has shown efficacy with a good side effect profile [7]. Canakinumab, an IL-1B receptor antagonist, was studied in the CONSIDER trial, which randomized 35 patients to treatment and placebo groups [10]. Treatment with canakinumab was not found to have statistically significant results compared to placebo, though a per-protocol analysis did have significant results. One caveat to this trial was the primary outcome measure, which was active joint involvement. The authors feel that this may not be representative because AOSD is a systemic disease. Tofacitinib, a JAK 1/3 inhibitor, blocks the proinflammatory effect of a wide range of cytokines by blocking a common downstream pathway [11]. It also suppresses macrophage activation and function. In this trial of 14 cases, seven patients achieved complete remission with decreased prednisone requirements. Of the biologics, canakinumab is the only FDA-approved treatment for AOSD, and it was approved in June of 2020. Our patient experienced good symptomatic and serologic response on steroids and currently is planned to start methotrexate therapy in the near future for its steroid-sparing effect.

## Conclusions

This case exemplifies the diagnostic uncertainty and workup that is often required in the diagnosis of AOSD. Of note, highly elevated ferritin is an important diagnostic clue, though the Yamaguchi criteria should be followed. Providers treating patients with recurrent vague inflammatory symptoms should consider AOSD as a differential diagnosis. Refractory patients can be treated with one of several biological therapies at the discretion of their rheumatologist.
